# Accuracy of a novel mixed reality surgical platform for total knee arthroplasty

**DOI:** 10.1186/s42836-025-00340-z

**Published:** 2025-10-28

**Authors:** H. John Cooper, Aaron Young, Jacob B. Brenza, Mike E. King, Winona L. Richey

**Affiliations:** 1 Department of Orthopedic Surgery, Columbia University Irving Medical Center, New York, NY 10032 USA; 2 PolarisAR, Richardson, TX 75082 USA

**Keywords:** Augmented reality, Mixed reality, Total knee arthroplasty, Head-mounted display, Surgical navigation, Computer-assisted surgery

## Abstract

**Background:**

Computer-assisted navigation has improved surgeons’ ability to achieve accurate implant placement in total knee arthroplasty (TKA). As technology evolves and new systems are introduced, it is imperative to evaluate their accuracy for achieving desired goals.

**Methods:**

This work evaluated the accuracy of a novel mixed reality surgical guidance platform that uses a head-mounted device to measure patient bony anatomy, quantify soft tissue balance, and provide quantitative resection guidance overlaid on the surgical field. Accuracy is evaluated in a cadaveric simulated use study and in a comprehensive evaluation of the tracking subsystem using the international ASTM standard F2554-22. Depth and angular errors are reported across eight knees for the proximal tibial, distal femoral, and posterior femoral resections by comparing platform-navigated resection metrics to caliper-measured resection depths and post-operative CT-measured angles. Analysis of the tracking subsystem investigated instrument localization error across the tracking volume, including rotational and positional extremes. Testing also extended the ASTM standard to include additional rotation tests and an evaluation procedure for planar accuracy metrics.

**Results:**

All cadaveric simulated use depth and angular absolute errors were below 2 mm and 2°, with 83% less than or equal to 1 mm and 1°. Absolute resection errors averaged 0.7 ± 0.4 mm and 0.6 ± 0.4° for depths and angles, respectively. The tracking subsystem localized over 99.5% of points with positional accuracy better than ± 2 mm and localized over 99.5% of planes with angular accuracy better than ± 1°. Average absolute tracking errors were sub-millimeter and sub-degree.

**Conclusion:**

This open and imageless platform for TKA surgical guidance requires only 13 landmarks with no additional equipment footprint, minimal tooling, and data overlaid holographically onto the surgical scene, while still providing a comprehensive set of metrics with state-of-the-art accuracy.

Video Abstract

**Supplementary Information:**

The online version contains supplementary material available at 10.1186/s42836-025-00340-z.

## Introduction

Success in total knee arthroplasty (TKA) relies on several key goals: accurate implant alignment, rotation, and sizing, as well as soft tissue balance, for the purpose of ultimately improving patient satisfaction and knee stability. A properly balanced knee has been shown to improve long-term patient satisfaction [1] and proper knee alignment affects implant durability and post-operative functionality [2, 3], though the ideal strategy for obtaining a balanced and aligned post-operative knee remains a topic of open discussion [2–4]. Regardless of the surgical philosophy chosen, each strategy for a properly balanced and aligned knee relies on technical goals of executing the proper bony resections, balancing soft tissue laxity, and properly sizing and aligning the implant.

Recent advancements in technology have improved the ability to measure and achieve some of these technical goals. By quantifying patient anatomy and then navigating surgeons to their planned resection planes, robotic and computer-assisted navigation systems have improved surgical precision in TKA [5–10]. These technologies have been shown to improve implant alignment [5–7], improve short-term post-operative range of motion [9, 11], and recent studies have demonstrated a benefit in medium- and short-term patient-reported outcome measures [6–8]. Though most studies report equivalent long-term patient outcomes when robotic-assisted TKA is compared to manual TKA, even studies that report no significant differences between robotic and manual approaches generally report reductions in outliers and improved radiographic outcomes [12–14].

However, robotic guidance is not without limitations. Robotic guidance requires a large permanent footprint in the operating room, even when not in use, with space requirements ranging from 0.5 m^2^ to over 1 m^2^, in addition to added instrument trays taking up valuable operating room real estate and resources for preparation, cleaning, and sterilization [5, 15, 16]. In addition to the footprint of the robots, their monitors, and their associated tracking cameras, these guidance approaches often require preoperative imaging that exposes the patient to additional radiation, and is not always covered by insurance [10]. Beyond this, most robotic systems are closed platforms, meaning they are only compatible with certain vendor implants [10]. This may limit a surgeon’s ability to choose the implant they perceive to be the best option for the patient, and the ability to adopt new implant technologies as evidence arises to support new mechanical designs or alignment strategies.

Robotics has improved the precision of TKA, but emerging mixed reality solutions offer potential improvements even beyond the scope of robotics. Iacono et al. analyzed a mixed reality navigation device that provided radiographic accuracy comparable to that of robotic systems [17]. However, by providing guidance information directly overlaid onto the field of view, mixed reality solutions reduce or remove the need to reference screens, increasing focus on the patient and reducing workflow interruptions, while also eliminating the space requirement for guidance displays [17, 18]. By performing all computations and instrument tracking with the head-mounted device, mixed reality solutions also eliminate the need for external trackers and cart computers. Initial accuracy results are limited, though promising [19, 20]. A major limitation of this existing mixed reality system is that the femur and tibia are not tracked simultaneously, preventing it from providing the same breadth of information as robotic systems. Without simultaneous tracking of both the femur and tibia, systems cannot provide important soft tissue laxity metrics that have major implications for pre-resection soft tissue balance assessment and post-resection validation of soft tissue balance, nor can they quantify range of motion or hip-knee-ankle alignment.

The goal of the current study is to quantify the accuracy of a novel TKA mixed reality surgical navigation platform. This imageless, footprint-less platform, comprised of a mixed reality headset and a small portfolio of mechanical tools, requires fewer than 15 landmarks to provide the surgeon with computed metrics describing the patient’s bony anatomy and soft tissue balance. These metrics allow the surgeon to alter their surgical plan intraoperatively and set planned resection depths and angles based on intraoperative feedback and intraoperative measurements of soft tissue laxity in medial and lateral compartments. The proprietary toolset allows the surgeon to navigate to their desired resection planes for distal femoral, posterior femoral, and proximal tibial cuts, while also quantifying gap balancing, range of motion, and hip-knee-ankle alignment. This work summarizes platform accuracy in cadaveric simulated use by evaluating navigated resection planes and summarizes tracking subsystem accuracy by evaluating the set of proprietary tracked tools.

## Materials and methods

This section introduces the mixed reality surgical platform and then describes accuracy evaluation in two main subsections: platform accuracy and tracking accuracy. Platform accuracy, or the overall ability to guide resections to the surgeon-selected locations, was evaluated in simulated use testing on cadaveric specimens. Tracking accuracy, or the platform’s ability to locate points and planes with the disposable tracked tools, was evaluated with a machined phantom in the context of intended use.

### Mixed reality surgical platform

**Fig. 1:**
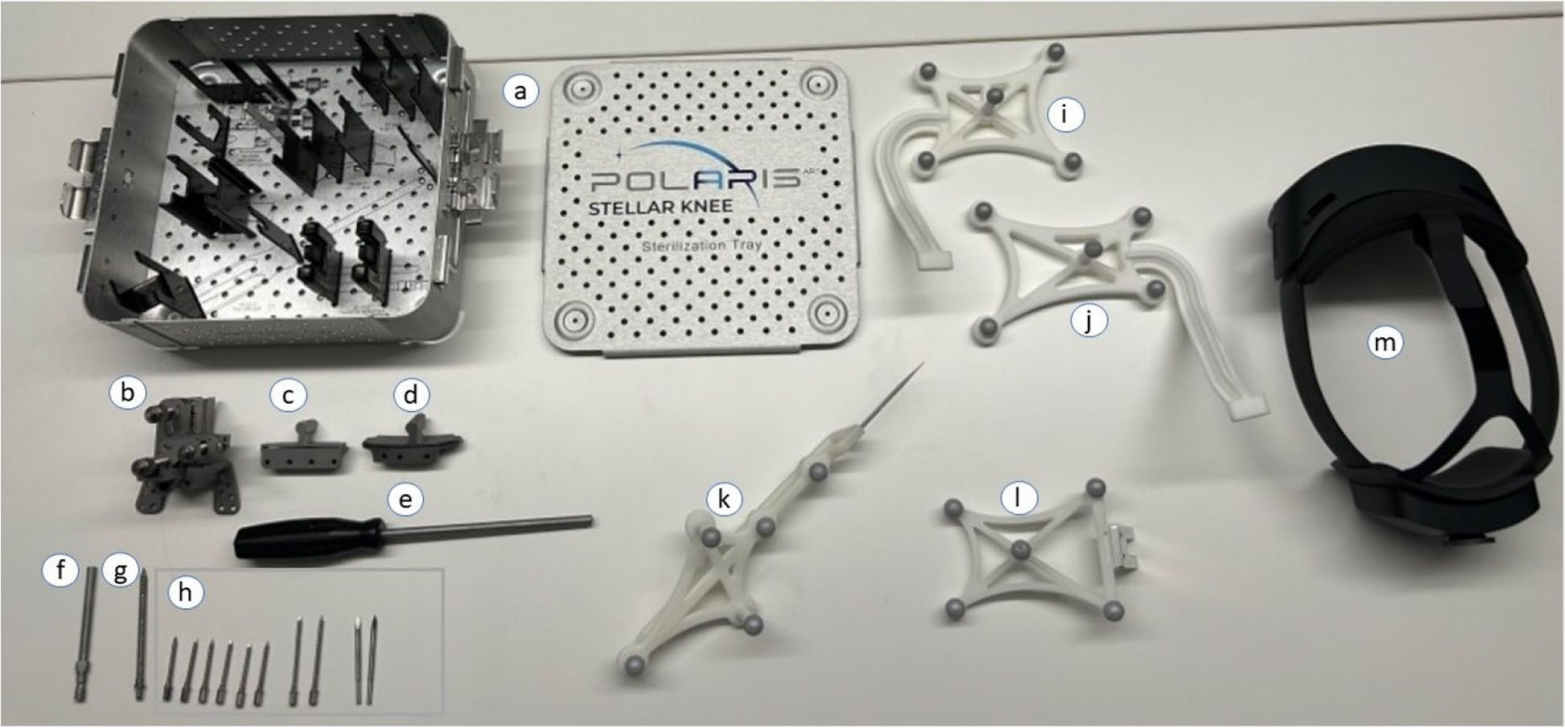
The complete hardware kit for this mixed reality surgical platform, including reusable instruments (a–g, m), disposable screws (h), and disposable tracked instruments (i–l) for a left TKA procedure. Instrumentation includes: (**a**) Sterilization Tray, (**b**) Universal Resection Guide, or URG, (**c**) Femoral Resection Block, (**d**) Left Tibial Resection Block, (**e**) Pin Driver, (**f**) Hudson adapter for power drilling headed pins, (**g**&**h**) Pins to secure hardware to bone, (**i**) Left Femoral Array, (**j**) Left Tibial Array, (**k**) Stylus, (**l**) Navigation Fin, (**m**) Head Mounted Device—Microsoft HoloLens 2

The mixed reality platform, STELLAR Knee (PolarisAR; Miami, FL, USA), guides TKA procedures from landmarking, through soft-tissue balance assessment, and femoral and tibial resection planning, execution, and validation. This FDA-cleared mixed reality surgical platform (Fig. 1) consists of a set of sterilizable reusable instrumentation, four disposable marker arrays, and a head-mounted device (HoloLens2, Microsoft; Redmond, Washington, USA). The Stylus is used for point-based data collection, and the Fin is used to navigate plane-based data collection, while the Femoral and Tibial Arrays serve as reference markers for intraoperatively measured femoral and tibial data, respectively.

**Fig. 2:**
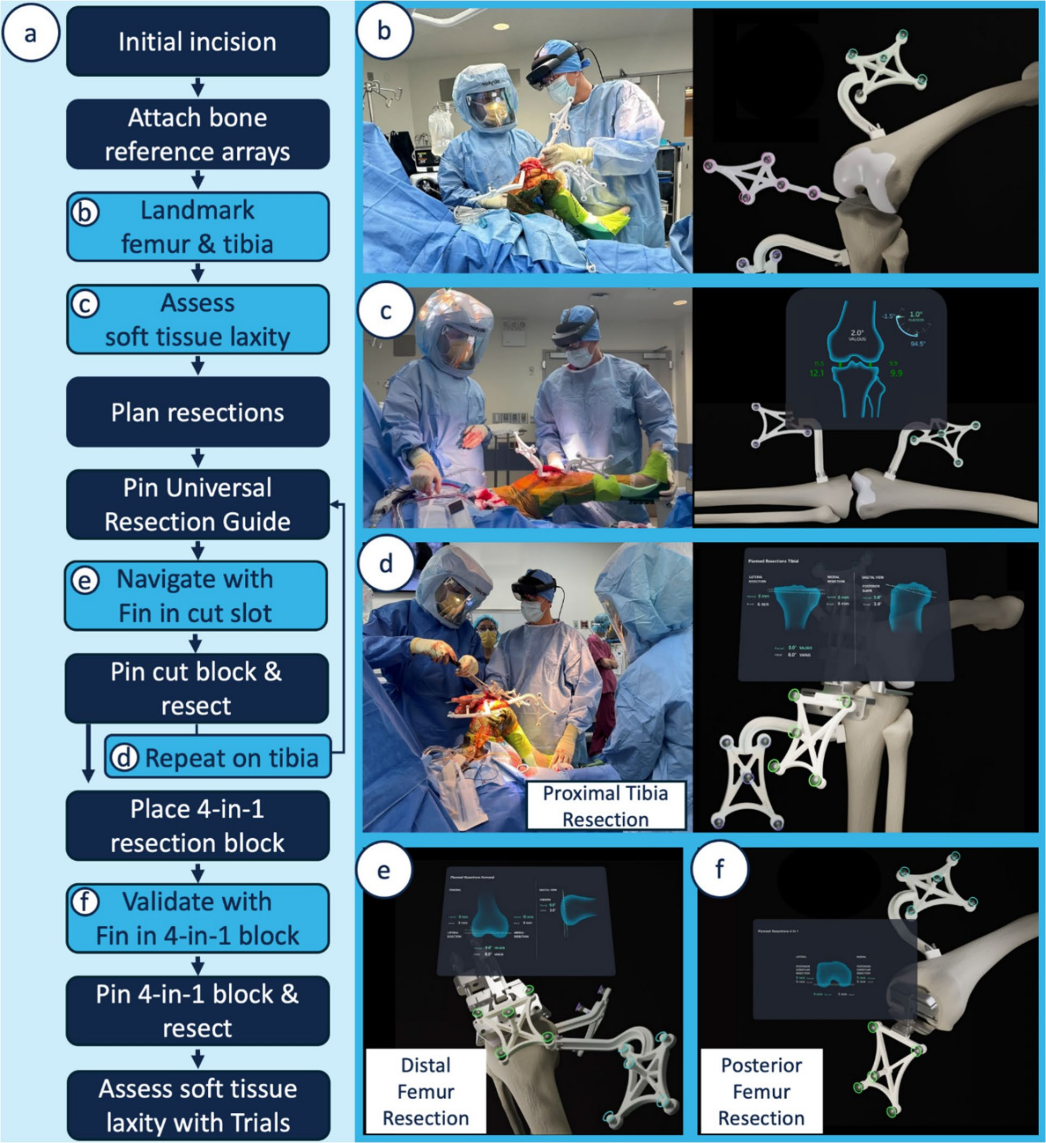
Workflow of the mixed reality platform for total knee arthroplasty. Overview (**a**). Key steps of landmarking (**b**), assessment (**c**), and navigation (**d**–**f**) are shown in an operating room setting as well as on a bone model with the augmented reality holographic content

The workflow of this mixed reality platform is summarized in Fig. 2. Reference arrays are attached intra-incisionally using two bone screws per array. Simultaneous tracking of the femur and tibia is enabled by the attachment configuration of the tracked arrays and the implementation of a sufficiently large tracking volume on the mixed reality headset. Landmarking requires 12 Stylus collected landmarks and the hip center. After landmarking, the platform provides gap information as the surgeon takes the knee through the range of motion and applies varus and valgus stresses. Next, a holographic intraoperative planning screen allows the surgeon to choose resection depths and planned gaps for the ideal soft tissue laxity. Intraoperative planning is optimized on a per-user basis by a set of surgeon preferences for metrics like tibial slope, femoral flexion, and varus/valgus angles for the tibia and femur. On a case-by-case basis, the angles start at the user-preferred values, and then angles and resection depths can be adjusted as desired. Once the surgeon is satisfied with the plan, the proprietary Universal Resection Guide (URG) is attached to the bone, and a proprietary cut block is fit onto the URG. Workflow allows starting with either the proximal tibial or distal femoral cut. The Fin is then inserted into the cut block’s saw slot, and a set of precision dials on the URG are precisely adjusted until the Fin-navigated plane aligns with the planned resections. The cut blocks are then pinned in place. After pinning, the Fin is re-inserted into the cut block for validation before resections. For the posterior femoral cut, the femur can be sized with the chosen implant system’s instruments. The Fin’s metal feet can be inserted into the posterior cut slot on an implant manufacturer’s 4-in-1 cut block to validate the posterior femoral resection plane.

**Fig. 3:**
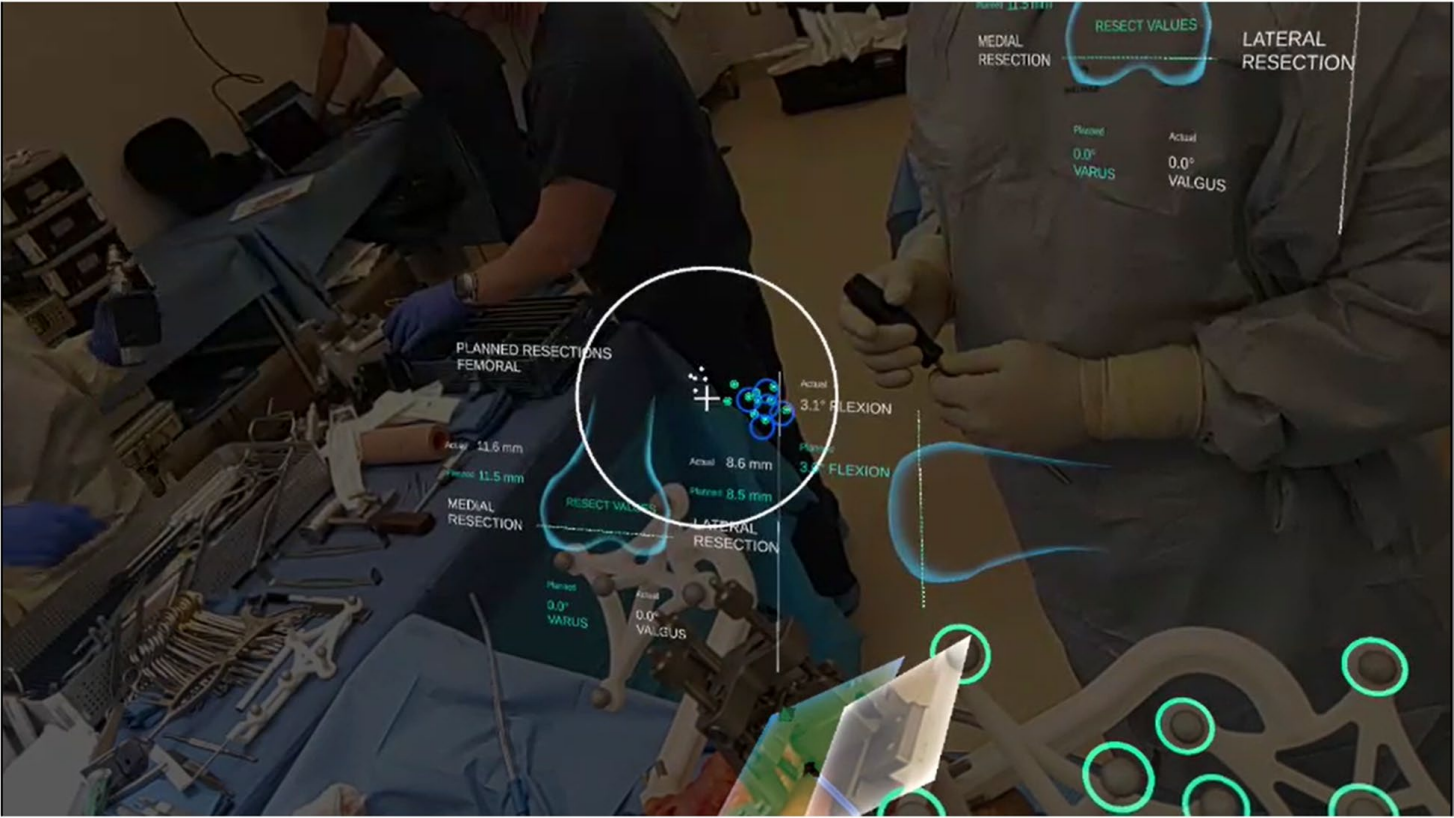
Surgeon’s view through the headset during a cadaveric procedure showing the alignment of the planned and navigated resection planes, shown in the center bottom as green and white planes, respectively. To the left of the holographic planes, the Universal Resection Guide is visible, attached to the distal femur. The white circle in the center of the view shows the reticle, a live display of the location of arrays within the field of view. The blue circles indicate the target position for the Femoral Array, and the small green circles show the live positions of the Fin and Femoral Arrays. The on-anatomy resection panel is visible behind the reticle, and in the top right of the image is a corner of the main display that can be moved and placed as desired

The platform guides the procedure by showing measured and computed data overlaid directly in the 3D environment (Fig. 3). During soft tissue balancing and resections, where the surgeon relies on the application for real-time feedback, the platform displays the pertinent metrics directly above the joint. This on-anatomy panel for resections is visible in Fig. 3. In addition to the on-anatomy panel, the platform features a main display panel, also visualized as a hologram in 3D space, that can be moved and placed freely in the room using built-in hand tracking and environment mapping. The surgeon controls the workflow with their choice of voice commands and/or holographic button presses on the main panel that maintain sterility without the need for draping screens or relying on assistance for platform interactions.

### Platform accuracy

Platform accuracy testing was performed in four pelvis-to-toe cadaveric specimens, where the left and right knees in each specimen were used for surgery for a total of eight cadaveric knees. As each side of the cadaver sample provides six linear and five angular measurements, these eight cadaveric knees provided 48 linear measurements and 40 angular measurements. The statistical results were computed using the formula for two-sided tolerance limits for normal distributions presented in [21], which accounts for the number of samples, $n$, using the scaling factor, $K$. The factor, $K$, is used to compute confidence intervals such that the probability is 95% that at least 90% of the distribution will be between $\overline{X }\pm Ks$, where $\overline{X }$ and $s$ are estimates of the mean and the standard deviation computed from a sample size of $n$. This study was performed by a single surgeon evaluator who was a contributor to the development of the product. After planning desired resections, the URG was used to pin the cut block in the desired resection plane. After pinning but before bone resections, cut block positioning was validated with the Fin and considered acceptable for all cases. Resections were then performed by the surgeon.

**Fig. 4:**
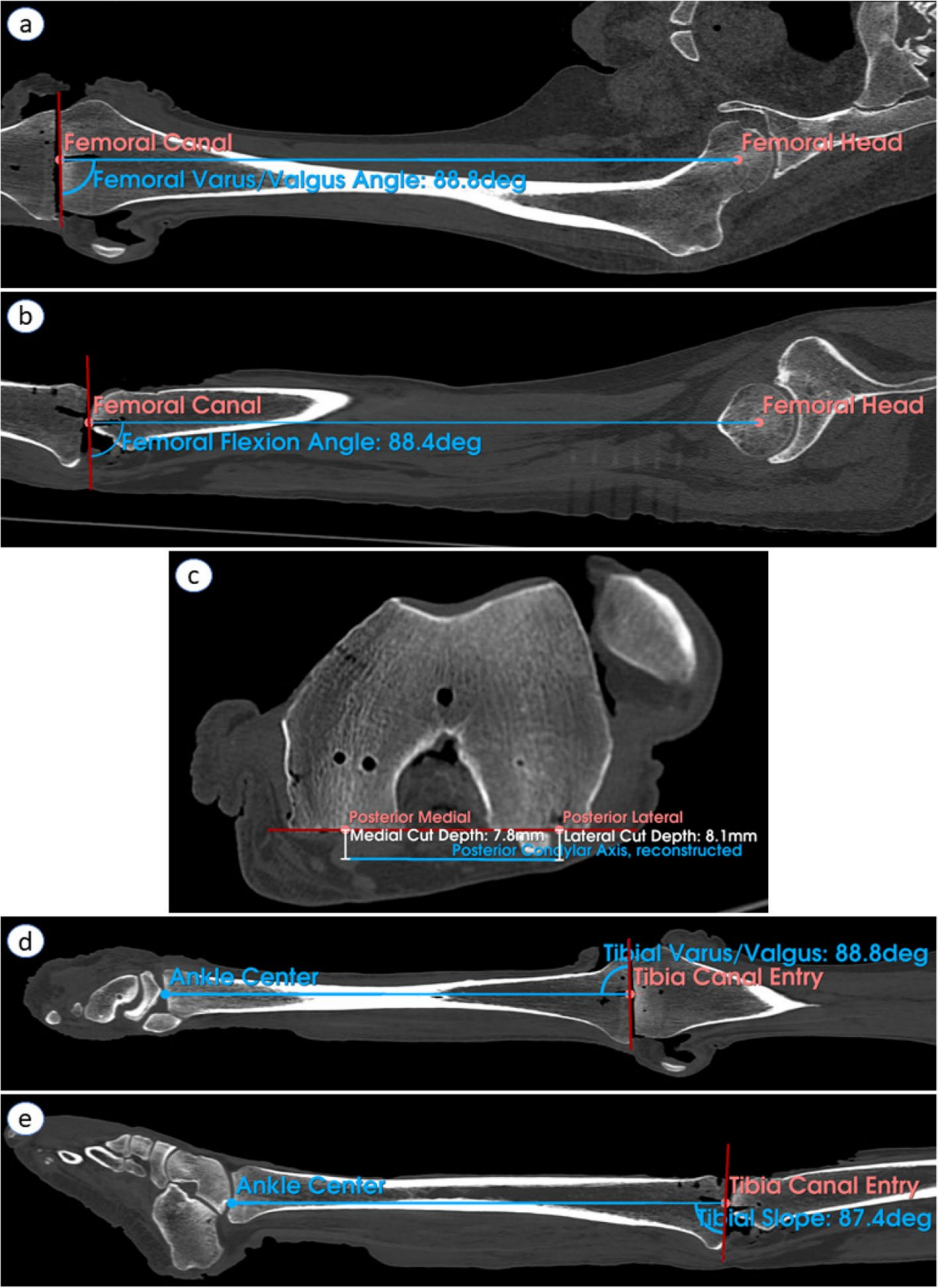
Angle computations on cadaveric post-operative CT (Left 1). Annotated landmarks are shown in coral, annotated resection planes are shown in red, and computed data are shown in blue. Caliper depth measurements are shown in white. Angles are computed between the blue anatomical axes and the red resection planes for (**a**) femoral varus/valgus angle, (**b**) femoral flexion angle, (**c**) femoral rotation, (**d**) tibial varus/valgus, and (**e**) tibial slope

Selected landmarks on the distal femoral condyles, posterior femoral condyles, and tibial sulci were marked with ink during landmarking for later caliper validation. Ground truth depth measurements to the nearest 0.1 mm were taken intraoperatively post-resection using calipers with a flat surface attachment on the resected plane and a point attachment on the selected landmark. The thickness of the saw was added to these caliper measurements. Ground truth angular measurements to the nearest 0.1° were taken from post-operative computed tomography (CT) images using anatomic landmarks (Fig. 4). Post-operative CT data were processed in 3D Slicer [22] and SolidWorks (Dassault Systèmes SolidWorks Corporation; Waltham, MA, USA). Ground truth measurements were compared to the surgeon-accepted metrics displayed by the platform prior to resection.

### Tracking accuracy

The ASTM F2554-22 standard, designed to evaluate point localization accuracy and repeatability in a surgical navigation system [23], was extended to be performed within the intended use of STELLAR Knee and to evaluate the plane tracking tool, the Fin. Tracking accuracy was evaluated on a machined metal phantom (per ASTM F2554-22) using a left STELLAR Knee disposable instrumentation kit. Testing was performed in an office setting under fluorescent lighting with noise from extraneous infrared reflective markers, computer screens, windows, aluminum foil, and metal objects.

Stylus testing evaluated point measurement precision and bias for phantom positions and orientations across the measurable volume with tool rotations along the three orthogonal axes. Generally, precision is evaluated by comparing platform-collected measurements to each other, and bias is evaluated by comparing platform-collected measurements to known dimensions of the phantom as measured by a coordinate measuring machine (CMM) and registered to the platform’s coordinate space. Briefly, Stylus testing as outlined in the ASTM standard includes Single Point Accuracy, Stylus Rotation, Point-to-Point Accuracy, Phantom (and therefore reference array) Rotation, and Phantom Locations. Stylus rotations included the full trackable range of motion as limited by line-of-sight and phantom divot geometry.

Similarly, Fin testing included analogous tests where six phantom surfaces were also measured by a CMM and registered to the platform’s coordinate space to provide ground truth data. Fin rotation is geometrically limited to be in-plane, and therefore, plane measurements were additionally collected across six phantom surfaces to capture a variety of tool angles and positions. Furthermore, the platform allows either surface of the Fin to be used for navigation. With the given phantom geometry, five of the six measured phantom surfaces were able to be evaluated using both sides of the Fin’s metal feet, capturing a comprehensive range of Fin rotations and view angles.

Both point and plane tracking evaluations were adapted and extended to consider the intended use of the platform. Since data from sufficiently different reference poses are never cross-compared in the intended use of STELLAR Knee, Phantom Rotation, and Phantom Locations testing (ASTM F-2554–22 Sects. 8.9 and 8.10 [23]) was performed in the context of platform constraints.

**Fig. 5:**
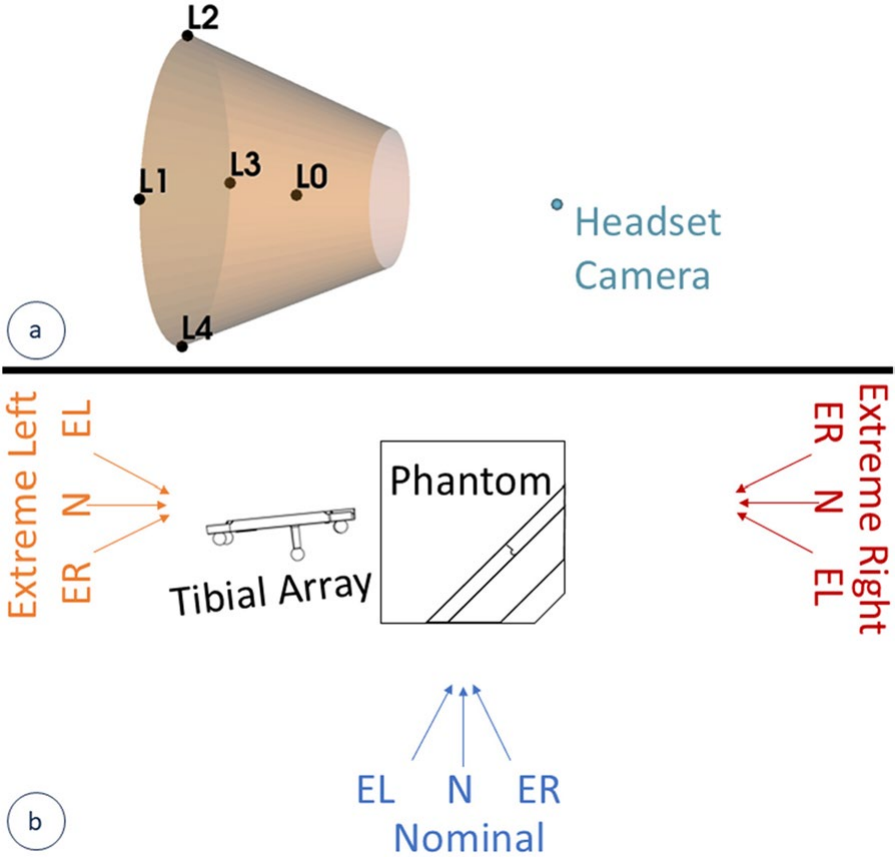
Diagrams of tracking accuracy testing for the variations in position (a) and rotation (b). **a** The headset’s tracking volume (orange) with respect to the headset’s camera (blue) and the five main locations where accuracy is evaluated (L0-L4), with L1-L4 representing positional extremes. **b** Reference rotation test view angles from a birds-eye perspective with the phantom and tibial array (note, tests are repeated for the femoral array). Each arrow represents a viewing angle where accuracy is evaluated. Phantom orientations are labeled, and phantom sub-views are abbreviated (EL = Extreme Left, N = Nominal, and ER = Extreme Right)

Phantom Location tests evaluated tracking reliability at all positions within the tracking volume, as shown in Fig. 5a. In keeping with the ASTM standard, the Phantom Location tests evaluated accuracy at five main positions in the trackable volume: Central (L0), Extreme Left (L1), Extreme Top (L2), Extreme Right (L3), Extreme Bottom (L4), where extreme locations represent the maximum trackable distance in two axes (e.g., Extreme Left is the maximum trackable position along the Z and X axes, depth and left/right directions respectively). For the Phantom Location test, the phantom registration to CMM ground truth data was performed only once at each of the five main locations. Though data from different main locations were not cross-compared, positional variations were added to the Phantom Rotation tests.

As opposed to Tool Rotation tests that kept the scene view static and varied the angle of the handheld tools (Stylus and Fin for the point and plane accuracy, respectively), the Phantom Rotation tests evaluated system robustness to viewing the Femoral or Tibial array from different angles. These tests were extended to collect data at all poses specified in the standard, as well as additional poses. Data were compared across multiple view angles and locations within each of the main view categories (Fig. 5b): (1) nominal view where the reference array was facing the tester, (2) extreme left view where the test setup was rotated to simulate the view from the foot of the patient with tools designed for a left knee, and (3) extreme right view where the test setup was rotated to simulate the view from the hip of the patient with tools designed for a left knee. For the Phantom Rotation tests, phantom registration was only performed once at each view category (once per color in Fig. 5b). Reference pose variation was quantified and available to the user during testing to ensure poses for each main view category spanned at least 40 degrees. Within each sub-view, the position of the array in the volume was varied slightly across 6 sublocations (not shown in Fig. 5b), where sub-L0 is the initial position and sub-L1 through sub-L4 represent positional extremes within the system’s visual cues. Since reference depth is not constrained visually, all view-angle analyses also included a worst-case sub-location (sub-L5) maximally far from the user before tracking was lost. All rotational and positional extremes were evaluated, but they were compared to the set of data in that local region using the visual cues present in the application (as shown in Fig. 3). In total, 54 combinations of view angles and locations, i.e., poses, were evaluated (3 main view categories, 3 sub-views, and 6 sub-locations).

## Results

### Platform accuracy

Depth and angular errors, as measured on the eight cadaveric knees, are reported in Tables 1 and 2. Depth errors averaged − 0.2 ± 0.8 mm (mean ± standard deviation) with a 95% confidence that 90% of data lies within the interval of [− 1.7 mm, 1.4 mm]. Angular errors averaged 0.0 ± 0.8° with a 95% confidence interval of [− 1.5°, 1.6°] at 90% reliability. Absolute errors averaged 0.7 ± 0.4 mm and 0.6 ± 0.4° for depths and angles, respectively. Example angle computations are visualized in Fig. 4.

**Table 1:** Cadaveric resection depth errors in millimeters

Measurement	Left 1	Right 1	Left 2	Right 2	Left 3	Right 3	Left 4	Right 4	*Mean*
Lateral Distal Femoral	− 0.4	− 0.8	− 0.9	− 1.0	− 0.3	− 0.5	− 0.4	− 0.7	* − 0.5*
Medial Distal Femoral	− 1.7	− 0.4	− 1.5	− 1.1	− 0.7	− 0.1	0.2	0.2	* − 0.6*
Lateral Posterior Femoral	0.4	0.9	0.6	− 0.2	0.6	0.4	0.2	0.5	* 0.4*
Medial Posterior Femoral	0.4	0.2	0.1	0.1	0.4	0.1	0.8	0.9	* 0.4*
Lateral Tibial	− 0.4	− 0.8	− 0.5	− 1.1	1.2	− 1.7	0.0	− 1.0	* − 0.5*
Medial Tibial	− 0.2	− 1.0	1.3	− 0.7	1.5	− 0.7	− 0.6	− 0.8	* − 0.2*

**Table 2:** Cadaveric resection angle errors in degrees. Positive varus/valgus angles indicate more varus rotation than planned, and positive femoral rotation angles indicate more internal rotation than planned

Measurement	Left 1	Right 1	Left 2	Right 2	Left 3	Right 3	Left 4	Right 4	*Mean*
Femoral Varus/Valgus Angle	− 1.1	0.7	− 1.4	0.2	− 0.5	0.3	− 1.0	0.0	* − 0.4*
Femoral Flexion Angle	− 0.3	− 0.2	− 0.1	− 1.0	0.3	1.3	− 1.5	− 0.8	* − 0.3*
Femoral Rotation Angle	− 0.1	0.3	0.5	− 0.3	0.7	0.1	− 0.7	0.9	* 0.2*
Tibial Varus/Valgus Angle	1.2	0.5	1.5	0.2	0.8	0.7	0.4	− 0.1	* 0.7*
Posterior Tibial Slope	− 0.1	0.0	0.6	0.6	0.9	− 0.2	− 1.5	− 0.4	* 0.0*

### Tracking accuracy

**Fig. 6:**
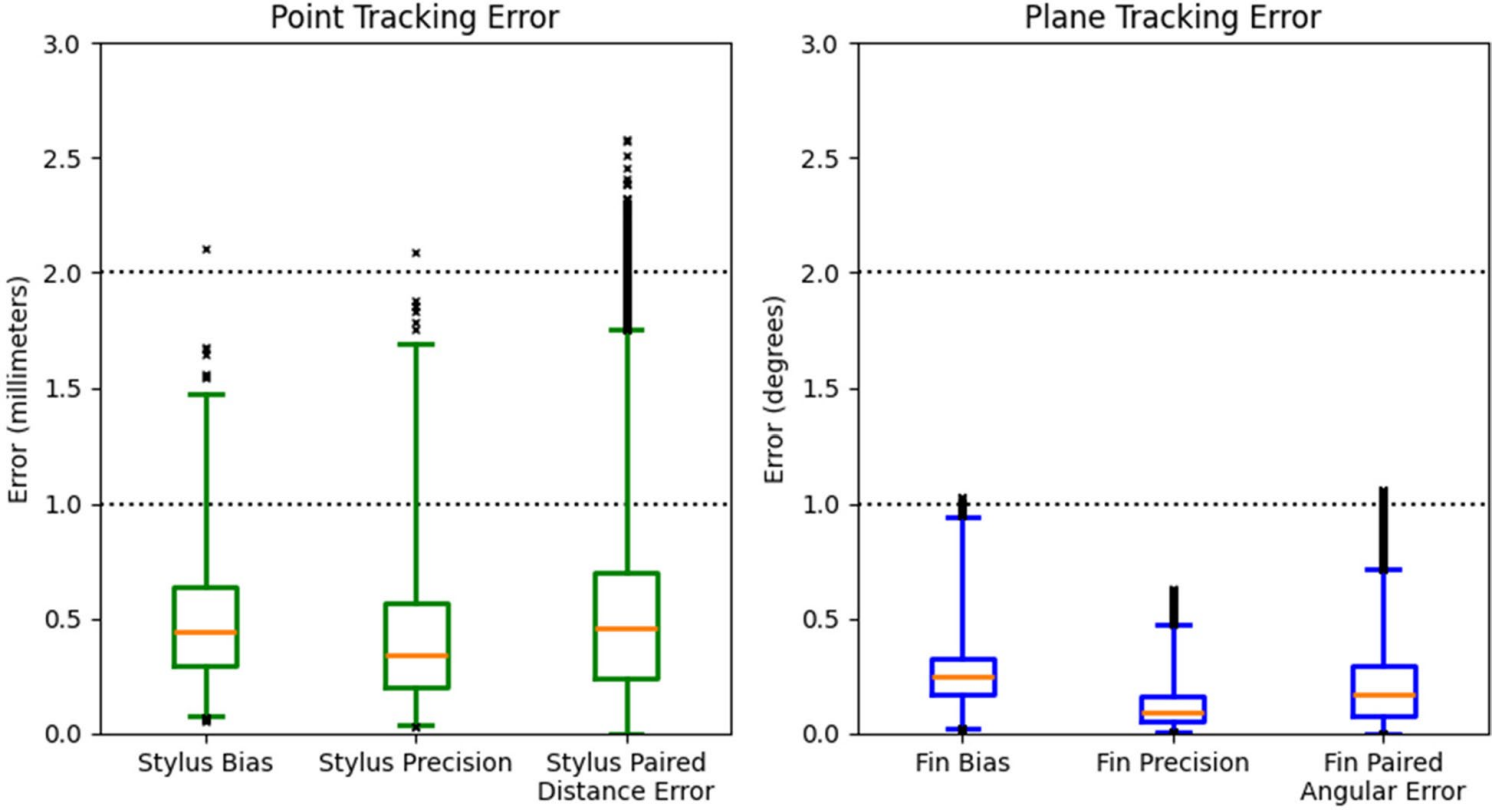
Absolute tracking error summarized across all ASTM data, where the central line (orange in color version available online) is the median, and the whisker caps show the 0.25 and 99.75 percentiles. Data outside this central 99.5% are plotted as “x” es

The results across all the extended ASTM-style tests (Fig. 6) demonstrated that the system can localize points with positional accuracy better than ± 2 mm and can localize planes with angular accuracy better than ± 1°. Point localization accuracy achieved 0.5 ± 0.3 mm bias (mean ± standard deviation) and 0.4 ± 0.3 mm precision, summarized across more than 2,700 points. Paired distance error, or inter-point distance error, was also 0.5 ± 0.3 mm, evaluated in over 395,000 combinations of Stylus measurements. Plane localization accuracy achieved 0.3 ± 0.1° bias and 0.1 ± 0.1° precision across more than 25,000 measurements. Paired angular error, or inter-plane angle error, was 0.1 ± 0.2° evaluated at over 13.9 million combinations of Fin measurements.

## Discussion

The cadaveric validation testing found that this novel, mixed reality surgical platform can navigate resection depths to within ± 2 mm and resection angles to within ± 2° of surgeon-selected planes. All in-vitro error metrics were less than 2 mm and 2°, with 83% of errors equal to or less than 1 mm and 1°. This level of accuracy for in-vitro data is on par with that of other currently available robotic and computer-aided TKA navigation systems [5, 7].

This technology compares favorably to currently available robotic and mixed reality systems, as shown by the accuracy comparisons presented in Table 3. It should also be noted that the authors in [24] did not use the instrumentation provided by the mixed reality guidance system. Instead, authors used a cut block provided by the implant manufacturer (Genus MB LS, Alder Ortho, Cormano, IT) that provided an additional physical check to validate the resection plane orientation. The authors don’t specify how often the cuts were adjusted based on this validation information external to the guidance system. Though many of the studies in Table 3 reported a higher number of outliers than the present study, they evaluated a larger number of knees than presented in this work, and some were performed on patients instead of cadavers, representing a more challenging evaluation environment.

**Table 3:** TKA navigation system accuracy comparisons of competitive robotic (VELYS, ROSA, and MAKO) and mixed reality (Knee +) systems

Navigation System	Study Design	Absolute Depth Error Results	Angular Results	Notes
VELYS (Depuy Synthes, Johnson & Johnson; West Chester, PA, USA)	40 cadaveric knees [25]	0.5–0.7 mm means	0.5°–1.5° means	Some errors > 3° and 3 mm
ROSA (ZimmerBiomet; Warsaw, IN, USA)	30 cadaveric knees [26]	63–93% ≤ 1 mm 93–100% ≤ 2 mm	63–93% ≤ 1° 87–100% ≤ 2°	Some errors > 3°
	14 cadaveric knees [27]	Means ≤ 1 mm	Means ≤ 1°, except 1.3° mean femoral flexion	Some errors > 3° and 3 mm
	86 patients [28]	n.d	Means ≤ 1°	Maximum ≤ 6°
MAKO (Stryker; Kalamazoo, MI, USA)	45 patients[29]	94% ≤ 1 mm	78% ≤ 1°	No errors > 3°
	96 patients [30]	n.d	Means ≤ 1°	22% > 3°
	29 patients[31]	n.d	71% ≤ 2°	13% > 3°
Knee + NexSight, Mixed reality (Pixee Medical; Besançon, France)	76 patients [24]	n.d	93% ≤ 1°	No errors > 2° Tibial varus and slope only
	18 patients [20]	n.d	Means ≤ 1°	11% > 3°
STELLAR Knee, Mixed reality (PolarisAR; Miami, FL, USA)	8 cadavers	83% ≤ 1 mm Means ≤ 1 mm	83% ≤ 1° Means ≤ 1°	No errors > 2° or 2 mm

The tracking subsystem testing showed that the mixed reality system can track points and planes with accuracy better than ± 2 mm and ± 1°. System-wide analyses that incorporate all the nuances of the ASTM test are sparse. Most robotic systems for TKA utilize optical trackers from Northern Digital Inc. (Northern Digital Inc., Waterloo, ON, Canada) for instrument localization. NDI reports high accuracy and precision for tracking an individual fiducial sphere. However, the accuracy of a complete system will be dependent on tool calibration, reproducibility of fiducial attachment to the instrument frame, and lever-arm error effects related to the use of a reference array. While NDI reports the accuracy of tracking a single point fiducial, comprehensive ASTM-style testing is limited for a stylus with a reference.

In a project sponsored by one of NDI’s competitors, Smith & Nephew (Memphis, TN, USA), NDI’s Polaris Spectra was evaluated using the ASTM standard F2554-18, released in 2018 [32]. While that analysis also shows that the NDI tracking accuracy degrades at the edges of the volume with many maximums over 2.5 mm and some over 4.5 mm, these large values are not necessarily directly comparable to the results presented here. Designed to be mounted on the edge of the surgical scene, the Spectra has a large tracking volume that makes it feasible to orient the tracker volume to be centered on the surgical scene. Comparatively, the STELLAR Knee platform tracks using the head-mounted device and has a conical tracking volume (depicted, not to scale, in Fig. 5a) with a maximum tracking depth less than 1 m. Though this design allows for a small profile that also alleviates some line-of-sight constraints, it means that the edges of this platform’s tracking volume may be used more often than NDI’s. When evaluated in the center of the tracking volume, NDI Spectra errors were on the order of 0.5 to 1.5 mm on average, with maximums on the order of 2 mm. Inter-point distances in the center of the NDI Spectra tracking volume were generally below 1 mm. In 2016, NDI released the Polaris Vega, which has recently replaced the Spectra in many navigation systems [33]. Though ASTM test results are unavailable for these newer trackers, the tracking presented here appears equivalent to other optical trackers on the market.

Recently, another mixed reality tracking subsystem, also using the Microsoft HoloLens 2, was evaluated using the ASTM F2554-18 standard [34]. Accuracy for point-tracking was on the order of 1–2 mm, though their system does not contain a reference array, and their testing did not cover multiple view angles or phantom locations within the trackable volume. Because the testing presented here covers many more test conditions and achieves similar accuracy, tracking with the STELLAR system is likely superior or equivalent to the approach presented in [34].

This study shows an impressive initial work towards a small footprint mixed reality system that allows live simultaneous tracking of both the femur and tibia; however, there are several limitations of this work. This study is, of course, limited by the relatively small number of knees and the data collection on cadavers with a single surgeon experienced with the system. Real surgical scenarios represent additional challenges not captured in cadaveric studies, including but not limited to lens obstruction by blood, joint mobility (e.g., differences in soft tissue laxity and pelvis instability), and environmental instability (e.g., anesthesia drapes and increased personnel movement). Future evaluations, already underway, will include a larger sampling of knees and, with recent FDA clearance of the STELLAR Knee System, data across initial patient populations. So far, the platform has been used successfully for over 250 patient cases. The study is also limited by its use of the implant manufacturer’s femoral sizer for drilling holes for the 4-in-1 cut block. In the cases presented here, the posterior femoral resections were validated with the platform’s Fin, and none required adjustment to achieve accurate results. Though the posterior femoral cut is not yet navigated with platform-provided equipment, platform-provided validation offers intraoperative confidence in the performed resections and allows for the possibility to reposition the 4-in-1 guide in the event of misalignment.

As surgical technologies continue to be developed and adopted, there is a growing need to reduce intraoperative clutter [35]. This system pushes the needle on dramatically reducing the size of navigation technology with respect to footprint—by eliminating screens, computer carts, machinery, and stand-alone instrument trackers—and also with respect to instrumentation—by reducing the guidance toolkits to one instrumentation tray and only four disposable tracked tools. Importantly, it does so without sacrificing accuracy or simultaneous tracking of the femur and tibia. From a workflow perspective, landmarking is similarly minimalistic without sacrificing accuracy or the number of available metrics, as the system quantifies soft tissue laxity, range of motion, hip-knee-ankle alignment, and provides guidance for the three main resections of TKA: distal femur, posterior femur, and proximal tibia. Similarly, the platform stays efficient and flexible by being imageless and open. This allows surgeons the flexibility to choose the implant and alignment technique they prefer and that best suits the patient. The platform keeps the saw in the hands of the surgeon, yet allows for precise navigation and control to obtain accurate and reliable resections without damaging the soft tissue envelope. Whether precise control of these variables can raise the ceiling on successful TKA procedures remains to be evaluated, but it has the potential to raise the floor substantially. With its portable nature and competitive price point, this system is more accessible than its large robotic competitors, making comprehensive and accurate navigation available to large and small surgical centers and surgeons of all case-load volumes.

## Conclusions

This work demonstrates that this novel mixed reality surgical platform is accurate and reliable in vitro, providing accuracy comparable to modern robotic-assisted TKA systems. All in-vitro error metrics were below 2 mm and 2°, with 83% of error metrics at or below 1 mm and 1°. The tracking subsystem, implemented on a head-mounted device, similarly performs exceptionally well. When evaluated across the tracking volume—at a central location and rotational and positional extremes—average tracking error was sub-millimetric and sub-degree. This open-platform, imageless system has a reduced footprint, reduced toolkit, and data overlaid holographically directly onto the surgical scene, while still providing a comprehensive set of metrics with state-of-the-art accuracy.

## Data Availability

The datasets used and/or analyzed during the current study are available from the corresponding author on reasonable request.

## Abbreviations

TKA: Total knee arthroplasty
CT: Computed tomography
URG: Universal Resection Guide
CMM: Coordinate measuring machine
